# Exosomes Secreted from Adipose-Derived Stem Cells Are a Potential Treatment Agent for Immune-Mediated Alopecia

**DOI:** 10.1155/2022/7471246

**Published:** 2022-02-03

**Authors:** Yanqiao Li, Guangxing Wang, Qian Wang, Yun Zhang, Lei Cui, Xin Huang

**Affiliations:** ^1^Department of Dermatology, Tongji Hospital, School of Medicine, Tongji University, Shanghai 200092, China; ^2^School of Medicine, Tongji University, Shanghai 200092, China; ^3^Institute for Regenerative Medicine & Department of Joint Surgery, Shanghai East Hospital, Tongji University School of Medicine, Shanghai 200092, China; ^4^Department of Plastic Surgery, Beijing Shijitan Hospital Affiliated to Capital Medical University, Beijing 100038, China; ^5^Department of Stem Cells and Regenerative Medicine, School of Medicine, Tongji University, Shanghai 200092, China; ^6^Key Laboratory of Spine and Spinal Cord Injury Repair and Regeneration, Ministry of Education of the People's Republic of China & Department of Orthopedics, Tongji Hospital, Tongji University School of Medicine, Shanghai 200092, China

## Abstract

**Background:**

Alopecia has become an exceedingly prevalent dermatological disorder. Etiologically, infection (bacterial and fungal infection), inflammation, and immune dysregulation are the main causes of immune-mediated hair loss. Treating hair loss has remained challenging as the available therapies are limited. Exosomes from adipose-derived stem cells (ADSC-Exos) have been used for treating neurodegenerative diseases and autoimmune diseases and in wound-healing treatments. However, the function and mechanism of ADSC-Exos in alopecia treatment remain unclear. This study is aimed at investigating the effects of ADSC-Exos on hair growth *in vitro* and *in vivo* for potentially treating immune-mediated alopecia and further exploring the underlying mechanism.

**Methods:**

Cell proliferation, migration, and apoptosis of dermal papilla cells (DPCs) that were treated with ADSC-Exos were detected using the cell counting kit-8 (CCK-8) assay, scratch wound-healing assay, and flow cytometry assay, respectively. A C57BL/6 hair-depilated mouse model was established *in vivo*; then, ADSC-Exos were subcutaneously injected alone or in combined with minoxidil. The effects of ADSC-Exos on hair growth, pathological changes, and the related mechanism were investigated by HE staining, quantitative real‐time PCR (qRT-PCR), western blotting, and RNA sequencing (RNA-seq).

**Results:**

ADSC-Exos significantly promoted DPC proliferation and migration while also reducing apoptosis. In addition, compared with the control group, ADSC-Exos-treated mice had better hair growth, more hair follicles (HFs) and thicker dermis. RNA-seq revealed that the miR-22 and TNF-*α* signaling pathways were markedly downregulated in DPCs after ADSC-Exos treatment. In addition, according to qRT-PCR and western blotting results, the Wnt/*β*-catenin signaling pathway was activated in the skin of ADSC-Exos-treated mice.

**Conclusion:**

ADSC-Exos therapy positively affected the promotion of hair regrowth by regulating miR-22, the Wnt/*β*-catenin signaling pathway, and the TNF-*α* signaling pathway, implying that ADSC-Exos could be a promising cell-free therapeutic strategy for immune-mediated alopecia.

## 1. Introduction

Alopecia, also known as hair loss, is a widespread dermatologic disorder characterized by shorter anagen and longer telogen phases in the hair cycle. Hair loss affects millions of people worldwide and can be devastating to an individual's psychoemotional well-being. Major triggers of hair loss include microbial dysbiosis of the scalp that is caused by infection and inflammation of the HFs and immune dysregulation [1], which can affect HF immunology, cycling, and regeneration [2]. Traditionally, treatment options for hair loss have been limited to systemic treatments and topical applications. Currently, only finasteride and minoxidil have been approved by the Food and Drug Administration (FDA) as medications for alopecia treatment [3]. Prolonged use of finasteride can have several adverse effects on sexual and reproductive health. For inflammatory and autoimmune hair diseases, such as alopecia areata, treatments also remain a challenge and are mainly aimed at containing symptoms by topical corticosteroid application and immunotherapy [4]. Topical medications have favorable side effects compared to those of systemic therapies, but many topical medicines are poorly absorbed transdermally, limiting their effective concentration at HFs and targeting dermal HFs. Other approaches, including prostaglandin F2*α* (PGF2*α*) analogs, Janus kinase (JAK) inhibitors, cytokine inhibitors [5], laser therapy [6], and microcurrent stimulation [7] still lack clinical trials that are evidence-based and at a high level to affirm their efficacy. Therefore, the available treatments are far from desirable.

Hair growth is widely recognized as a result of the periodic remodeling of HFs [8]. Within the remodeling duration, DPCs, specialized mesenchymal cells that localize beneath the bottom of HFs, play key roles in the initiation of HF growth in anagen [9]. More recently, mesenchymal stem cells (MSCs) exhibited potential for hair regeneration. It was reported that adipose-derived stem cells (ADSCs) appeared to facilitate hair growth by modulating the DPC cell cycle and promoting DPC proliferation [10]. However, the clinical application of stem cells is still limited due to the low viability of implanted cells, the underlying risk of immunological rejection and tumorigenesis, and ethical restrictions. To address the clinical demands for hair loss treatment, developing a cell-free agent with improved efficacy and safety is essential.

A growing number of studies have found that exosomes, which act as a cell-free agent, may play an important role in alopecia treatment. Exosomes are nanoscale extracellular vesicles (EVs) originating from endosomes with a size range from 40 to 160 nm in diameter [11]. As important carriers of signaling molecules in intercellular communication, exosomes are being developed as potential therapeutic agents or prognostic biomarkers in multiple disease models [12, 13]. Previous studies have demonstrated that ADSC-Exos can be used for wound healing, atopic dermatitis, cardiovascular, inflammatory, and autoimmune diseases [14]. It has been shown that DP spheroid-derived exosomal miR-218-5p can promote hair regeneration and upregulate the *β*-catenin signaling pathway [15]. At present, few studies have explored the effects of ADSC-Exos on hair regrowth. The only example comes from Wu et al. [16], who demonstrated that ADSC-Exos were beneficial to the hair growth of transplanted hair in nude mice with increased growth factors such as platelet-derived growth factor (PDGF) and vascular endothelial growth factor (VEGF) and decreased TGF-*β*1 in skin tissue. However, the molecular mechanism by which ADSC-Exos promote hair growth has not yet been elucidated, and more work needs to be performed.

In this study, we aimed to comprehensively investigate the effects of ADSC-Exos on promoting hair growth and the underlying mechanism *in vitro* and *in vivo* for prospective treatment of immune-mediated alopecia. We revealed for the first time that ADSC-Exos could promote the proliferation, migration, and apoptosis inhibition of DPCs *in vitro*. In particular, we developed a C57BL/6 hair-depilated mouse model in which mice were subcutaneously injected with ADSC-Exos with or without a combination of topical minoxidil. Through RNA-seq analyses, we identified miR-22, a newly discovered key posttranscriptional regulator of the hair cycle, as a downstream target of ADSC-Exos in DPCs. The Wnt/*β*-catenin pathway was also shown to play a role in ADSC-Exos-induced hair regeneration. This study provides the potential for developing ADSC-Exos as a cell-free therapeutic strategy for immune-mediated hair loss in the future.

## 2. Materials and Methods

### 2.1. Participants

In this study, we gathered several representative cases of scalp infections, inflammation, and autoimmune illnesses, including bacterial and fungal scalp infections, seborrheic dermatitis, and alopecia areata, all of which can eventually result in temporary or permanent hair loss. The study was conducted in accordance with the Declaration of Helsinki. Patient diagnoses were made based on clinical manifestations with or without etiological examination.

### 2.2. Animals

All animal experiments were performed in compliance with institutional guidelines and were approved by the Animal Research Committee of Tongji University (TJAA07021401). Specific pathogen-free (SPF) Sprague–Dawley (SD) rats (male, 60–80 g) and C57BL/6 mice (male, 7 weeks or 7 days old) were purchased from Shanghai Slac Laboratory Animal Co., Ltd. (Shanghai, China). All animals were kept in a constant temperature environment (22-24°C) with a 12 h light/dark cycle. Animals were euthanized or grouped according to the experimental design for the following procedure.

### 2.3. ADSC Culture and Identification

ADSCs were harvested and isolated as previously described [17]. In brief, ADSCs isolated from the inguinal adipose tissues of C57BL/6 mice (7 days old) were cultured in Dulbecco's modified Eagle's medium (DMEM) containing 10% FBS (Gibco, USA) at 37°C and 5% CO_2_. Characterization of ADSCs was performed by detecting their multilineage differentiation potential. For adipogenic differentiation, ADSCs were incubated with adipogenic differentiation medium (Cyagen Biosciences Inc., China) for 2 weeks and stained with Oil Red O. For osteogenic differentiation, ADSCs were incubated with DMEM supplemented with 10% FBS, 100 nM dexamethasone (Sigma Aldrich, USA), 50 *μ*M ascorbate-2-phosphate (Sigma Aldrich, USA), and 10 mM *β*-glycerol phosphate (Sigma Aldrich, USA) for 3 weeks and were stained with Alizarin Red S. For chondrogenic differentiation, the micromass of ADSCs was incubated with chondrogenic differentiation medium (Cyagen Biosciences Inc., China) for 3 weeks and stained with Alcian Blue.

### 2.4. Isolation and Identification of ADSC-Exos

ADSCs at passage 3 were cultured for 48 h in DMEM containing 10% exosome-depleted FBS, which was obtained by ultracentrifugation at 120,000 × g for 18 h. The cell culture supernatant was collected by concentrating the conditioned medium in 100 K MWCO Amicon®Ultra15 Centrifugal Filter Devices (Millipore, USA). Exosomes were isolated using ExoQuick-TC (SBI Biosciences) at a ratio of 1 : 5 (ExoQuick-TC: supernatant) according to the manufacturer's instructions [18], resuspended in 200 *μ*L PBS, and stored at −80°C for subsequent experiments. The exosome protein levels were quantified with a Pierce BCA Protein Assay Kit (Thermo Fisher Scientific, USA). Exosomes were characterized by examining the particle size, morphology, and protein markers based on MISEV2018 minimal information proposed by the International Society for Extracellular Vesicles (ISEV) [19]. The size distribution of exosomes was analyzed by nanoparticle tracking analysis (NTA) using a ZetaView PMX 110 (Particle Metrix, Meerbusch, Germany). The morphology of exosomes was observed by transmission electron microscopy (JEM-1230; JEOL, Japan). Markers of exosomes, including TSG101, CD9, and CD81, were verified by western blot analysis.

### 2.5. Preparation and Identification of DPCs

The primary DPCs were harvested according to the needle microdissection procedure as previously described with a small modification [20]. Briefly, dermal papilla (DP) was isolated using 25G syringe needles from individual hair follicle (HF) that was harvested from rat whiskers under an Olympus stereomicroscope. The released DP was collected and cultured in DMEM supplemented with 20% FBS and 1% penicillin/streptomycin. On day 10, the primary DPCs almost completely migrated out of the DP condensates and were detached with 0.05% trypsin/EDTA (Gibco, USA) and collected by centrifugation. DPCs were maintained in DMEM supplemented with 10% FBS and 1% penicillin/streptomycin, and DPCs at passages 3-5 were used in subsequent experiments. For DPC identification, cells at passage 3 were stained for the presence of alkaline phosphatase（ALP） with a BCIP/NBT Alkaline Phosphatase Color Development Kit (Absin, China) according to the manufacturer's instructions. Immunofluorescence staining was performed to detect the expression of *α*-SMA (1 : 1000 dilution; Boster, China) or Nestin (1 : 200 dilution; Abcam, USA). The expression of the specific marker *SOX2* was detected by qRT-PCR. The primers used were presented in Table S1.

### 2.6. Exosome Labeling and Uptake Assay by DPCs

Exosomes were labeled with the red fluorescent dye Dil kit (Beyotime, China) for tracking *in vitro*. Then, Dil-labeled exosomes at final concentrations of 100 *μ*g/mL, 200 *μ*g/mL, and 400 *μ*g/mL and PBS were cocultured with DPCs (2 × 10^5^ cells/well) in DMEM containing 10% exosome-depleted FBS for 48 h. After 48 h, the culture medium was discarded, and the cells were washed with PBS 3 times. Then, the DPCs were stained with the green fluorescent dye Dio kit (Beyotime, China) according to the instructions. After being incubated for 10 min in dye working solution, the reaction was terminated by removing dye and washing cells with PBS 3 times. Uptake was observed under an Olympus fluorescence microscope (BX53, OLYMPUS, Japan).

### 2.7. Cell Proliferation, Scratch Wound-Healing Assays, and Flow Cytometry Analysis

DPCs were seeded in 96-well plates (Corning Costar, Brumath, France) at 5 × 10^3^ cells per well. After an overnight incubation, the culture medium was pipetted out, and ADSC-Exos were added, reaching final concentrations of 50, 100, 200, and 400 *μ*g/mL. Cocultures of DPCs and ADSC-Exos were maintained in 10% exosome-depleted FBS medium for 24, 48, and 72 h. Cellular proliferation was measured by the cell counting kit-8 (CCK8; 1 : 10, Dojindo, Japan) at 450 nm wavelength using a SpectraMax M5 plate reader (Molecular Device). Cell migration was assessed by the scratch wound-healing method. When passaged DPCs reached nearly 95% confluency, a scratched wound was made using a sterile 200 *μ*L pipette tip. The medium was replaced with FBS-free medium containing ADSC-Exos (100, 200, and 400 *μ*g/mL). Images of cell migration within the wounded area were captured at 0, 24, 48, and 72 h. The percentage of the healing area to the entire wound was determined using the ImageJ software (NIH, Bethesda, MD, USA). To induce apoptosis in DPCs, cells were exposed to 600 *μ*M hydrogen peroxide (H_2_O_2_) diluted with serum-free DMEM for 2 h. H_2_O_2_ stock solution (3%) was purchased from Sigma. Following treatment with ADSC-Exos (0, 100, and 200 *μ*g/mL) dissolved in 10% exosome-depleted FBS medium for 48 h, DPC apoptosis was determined by Annexin V-FITC apoptosis detection kits (Beyotime, China) and analyzed by a CytoFLEX flow cytometer (Beckman Coulter, USA).

### 2.8. In Vivo Hair Growth Experiments in C57BL/6 Mice

To investigate the effect of ADSC-Exos alone on hair growth, mice were randomly divided into 3 groups: the blank group (*n* = 5), PBS group (*n* = 5), and ADSC-Exos group (*n* = 5). Mouse dorsal hair was removed from a 2 cm × 2 cm area with an electric shaver at day 0. Then, each mouse in the PBS group received 250 *μ*L of sterile PBS (50 *μ*L per site, 5 injection sites) subcutaneously injected once into the dehaired area. The ADSC-Exos group received ADSC-Exos (400 *μ*g/per mouse, 400 *μ*g/EA, and 50 *μ*L per site, 5 injection sites) subcutaneously injected once into the dehaired area, whereas the blank group received no treatment as a blank control. The 5 injection spots were evenly distributed on the dorsal skin and spaced approximately 1 cm apart. The mice were sacrificed at day 20, and dorsal skin samples were harvested for further analysis. To explore the efficacy of ADSC-Exos in combination with 5% minoxidil (MNX), mice were divided into the MNX group (*n* = 5), PBS+MNX group (*n* = 5), and ADSC-Exos+MNX group (*n* = 5). Due to the skin irritation that possibly occurs from minoxidil to newly molted skin, treatments were performed 1 day later. MNX was topically applied once daily (100 *μ*L/per mouse) for 14 days. PBS (25 *μ*L per site, 9 sites) and ADSC-Exos (400 *μ*g/EA, 25 *μ*L per site, 9 sites) were administered once every 2 days for 14 days, and these were performed before minoxidil. The 9 injection sites were evenly distributed and separated by approximately 0.5 cm. Hair growth within 21 days was recorded photographically, and skin tissue was also collected on day 21 for further analysis.

### 2.9. Histological Analysis

The skin tissue was fixed in 4% paraformaldehyde, embedded in paraffin blocks, and then prepared for longitudinal or transverse sections of HFs at 5 *μ*m thickness, followed by HE staining. The HF numbers were calculated by the ImageJ software. The thickness of the dermis was measured as the distance from the dermal-epidermal junction to the juncture between the dermis and subcutaneous fat under a light microscope.

### 2.10. RNA Sequencing and Bioinformatic Analysis

For RNA-seq, DPCs were treated with PBS or 400 *μ*g/mL ADSC-Exos (DPC vs. Exo_DPC) for 48 h and harvested. Total RNA was isolated with TRIzol® reagent (Invitrogen, USA), and RNA quality was assessed using a BioAnalyzer 2100 system (Agilent). The library was sequenced with an Illumina HiSeq2500/4000 system. The differentially expressed genes were filtered based on the criteria of *q* value < 0.05 and ∣FoldChange | >2 for heatmap visualization. Kyoto Encyclopedia of Genes and Genomes (KEGG) pathway enrichment analyses were performed with the igraph R package clusterProfiler.

### 2.11. Quantitative Real-Time PCR

After collecting skin samples, excised skin specimens were immediately snap-frozen in liquid nitrogen and pulverized in precooled TRIzol using a tissue homogenizer (KZ-5F-3D, Servicebio). RNA was extracted using TRIzol, and reverse transcription and qRT-PCR were performed using a PrimeScript RT reagent kit and SYBR Premix Ex Taq (Takara, Japan). The data were normalized to the expression of *β*-actin. The expression of miR-22 was validated using a SYBR Green miRNA-based assay, and U6 was used as the standardized internal reference. Relative mRNA expression was calculated by the comparative 2^−*ΔΔ*Ct^ method.

### 2.12. Western Blotting Analysis

The exosomes were lysed in lysis buffer supplemented with protease inhibitor cocktail and phosphatase inhibitor cocktails. The kin samples were pulverized in cell lysates, and the supernatants were collected. Samples were loaded in 12% SDS–PAGE gels for electrophoresis (Bio–Rad, USA). The protein was transferred onto a PVDF membrane (Millipore) and incubated overnight at 4°C with primary antibodies against TSG101 (1 : 1,000 diluted, Abcam ab125011, UK), CD9 (1 : 1,000 diluted, Abcam ab92726, UK), CD81 (1 : 1,000 diluted, Abcam ab109201, UK), and *β*-catenin (1 : 2,000 diluted, Proteintech 17565-1-AP, USA). *β*-Actin served as the control. After incubation with HRP-conjugated secondary antibodies, the bands were visualized with SuperSignal West Pico Chemiluminescent Substrate (Thermo Fisher Scientific, USA).

### 2.13. Statistical Analysis

All data were presented as the means ± SD. The GraphPad Prism 8 software (USA) and ImageJ software were used for data analysis. Two-tailed unpaired Student's *t*-tests were employed for data statistics. A *P* value < 0.05 was considered statistically significant. ^∗^*P* < 0.05, ^∗∗^*P* < 0.01, ^∗∗∗^*P* < 0.001, and ^∗∗∗∗^*P* < 0.0001. ^####^*P* < 0.0001.

## 3. Results

### 3.1. Clinical Characteristics of Participants

**Figure 1:**
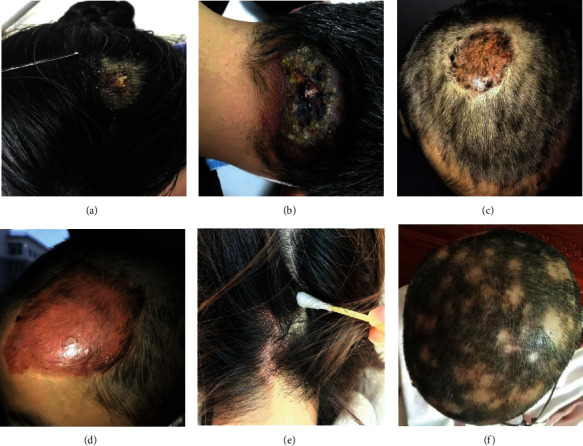
Clinical manifestations of infectious (bacterial and fungal infection), inflammatory, and autoimmune scalp diseases in immune-mediated alopecia are displayed. Bacterial infection: (a) scalp abscess and (b) scalp carbuncles. Fungal infection: (c) tinea capitis on top of the head and (d) tinea capitis in the forehead of the scalp. Inflammatory scalp disorder: (e) seborrheic dermatitis. Autoimmune hair loss: (f) alopecia areata.

Immune-mediated alopecia can be attributed to infection, inflammation, and autoimmune dysregulation. The clinical characteristics of patients with infectious, inflammatory, and autoimmune scalp disorders are presented in Figure 1. Patients exhibited bacterially infectious scalp disorder with abscesses (Figure 1(a)) and carbuncles (Figure 1(b)), fungally infectious scalp disorder with tinea capitis (Figures 1(c) and 1(d)), inflammatory scalp disorder with seborrheic dermatitis (Figure 1(e)), and autoimmune scalp disorder with alopecia areata (Figure 1(f)). Among these infectious scalp diseases, pathogenic tests showed a high detection rate of *Staphylococcus aureus*, which often led to septic scalp infections. If not treated promptly and effectively, all of these infectious, inflammatory, and autoimmune scalp illnesses mediated by the immune disorder can lead to scarring, nonscarring, and even permanent hair loss.

### 3.2. Successful Isolation and Characterization of ADSCs and ADSC-Exos

**Figure 2:**
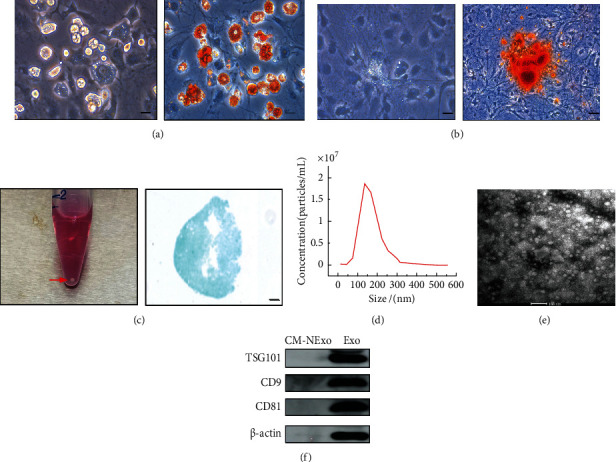
Characterization of ADSCs and ADSC-Exos. (a) Adipogenic differentiation of ADSCs was confirmed by Oil Red O staining. Scale bar = 100 *μ*m. (b) Osteogenic differentiation of ADSCs was confirmed by Alizarin Red S staining. Scale bar = 50 *μ*m. (c) Chondrogenic differentiation of ADSCs was confirmed by Alcian Blue staining. The red arrow indicates the micromass of ADSCs. Scale bar = 100 *μ*m. (d) Nanoparticle size distribution of ADSC-Exos was analyzed by NTA. (e) The morphology of ADSC-Exos was observed by TEM. Scale bar = 100 nm. (f) Western blotting was used to identify exosome-specific markers (TSG101, CD9, and CD81). CM-NExo: exosome-depleted culture medium; Exo: ADSC-Exos.

First, we aimed to isolate ADSCs from inguinal adipose tissue of mice, and characterization of ADSCs was performed by detecting the multilineage differentiation potential of adipogenic, osteogenic, and chondrogenic differentiation. ADSC-Exos were extracted from conditioned medium of ADSCs, and then, NTA, transmission electron microscopy (TEM), and western blotting were used to identify the ADSC-Exos. As a result, ADSCs were successfully isolated and cultured to the third generation. For adipogenic induction, lipid droplets were visualized by Oil Red O staining (Figure 2(a)). For osteogenic induction, calcium deposition was observed by Alizarin Red staining (Figure 2(b)). For chondrogenic induction, the formation of chondrogenic nodules was visualized by Alcian Blue staining (Figure 2(c)). The results demonstrated that the isolated cells were consistent with typical cell characteristics of ADSCs. Furthermore, exosomes were successfully extracted from conditioned medium of ADSCs. NTA showed that the average particle size of exosomes was 144.3 ± 57.7 nm (Figure 2(d)). TEM identified exosomes with a round and oval vesicle-like morphology (Figure 2(e)). Western blotting results verified that the exosome-specific markers TSG101, CD9, and CD81 were positive in ADSC-Exos but not in exosome-depleted culture medium (Figure 2(f)). These results indicated that ADSC-Exos were successfully isolated from conditioned medium of ADSCs.

### 3.3. DPC Preparation and Identification

**Figure 3:**
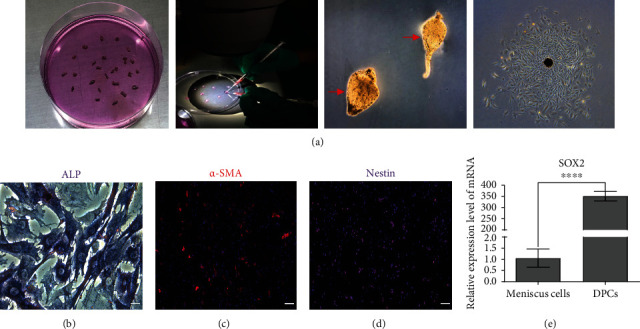
Preparation and identification of DPCs. (a) An individual whisker HF was isolated, and the DP was separated from isolated HF under stereomicroscopy. Red arrows showed the separated DP condensates. Primary DPCs gradually migrated from the DP condensates after incubation, and DPCs were successfully harvested. (b) ALP staining. Scale bar = 50 *μ*m. (c) Immunofluorescent staining of *α*-SMA. Scale bar = 100 *μ*m. (d) Immunofluorescent staining of Nestin. Scale bar = 100 *μ*m. (e) Expression of *SOX2* in DPCs was detected by qRT-PCR, and rat meniscus cells were used as a control.

DPCs play a key role in influencing the development of HFs and often act as a cellular model for alopecia studies *in vitro*. The isolated DP was garlic shaped, and primary DPCs were observed to migrate out around the DP condensates by day 3 (Figure 3(a)). Approximately 10 days later, DPCs were used for expansion until third- to fifth-generation cells were acquired. The identification results indicated that cell staining was positive for ALP (Figure 3(b)), *α*-SMA (Figure 3(c)) and Nestin (Figure 3(d)). *SOX2* was significantly expressed in DPCs by qRT-PCR (Figure 3(e)). Generally, DPCs exhibit typical DP cell characteristics.

### 3.4. Uptake of ADSC-Exos into DPCs Promoted DPC Proliferation and Migration and Alleviated H_2_O_2_-Induced Apoptosis

**Figure 4:**
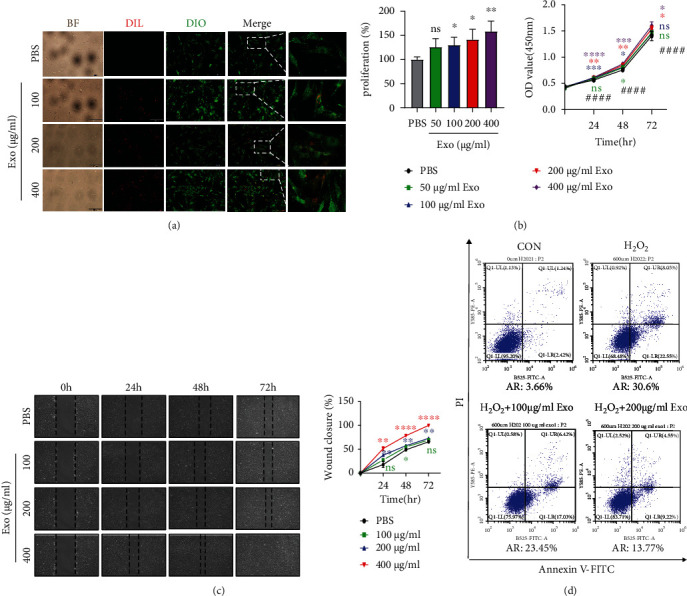
ADSC-Exos were taken up by DPCs and promoted DPC proliferation, migration, and apoptosis inhibition. (a) The internalization of Dil-labeled ADSC-Exos (red) by Dio-labeled DPCs (green) was observed. Bright-field (BF) images were also shown. Scale bar = 200 *μ*m. (b) DPC proliferation was assessed by CCK-8 assay. Data are expressed as the mean ± SD. *n* ≥ 3; ∗ means *P* value vs. the PBS group, # means *P* value vs. the 0 h group. (c) Scratch wound-healing assay. (d) Apoptosis flow cytometry assay. AR: apoptosis rate; Exo: ADSC-Exos. The experiment was repeated three times independently.

Then, we attempted to explore the interaction between ADSC-Exos and DPCs and the effect of ADSC-Exos on the biological function of DPCs in proliferation, migration, and apoptosis. For the uptake assay, the results revealed that ADSC-Exos were absorbed into the DPC cell membrane and further internalized inside the DPCs in a dose-dependent manner (Figure 4(a)). After being treated with ADSC-Exos for 48 h, DPC proliferation was dose-dependently increased in the ADSC-Exos group compared with the PBS group, and the cell number in the 400 *μ*g/mL ADSC-Exos group was significantly greater than that in the other groups (^∗^*P* < 0.001). The exosome treatment at 24 h, 48 h, and 72 h also indicated significant DPC proliferation compared with that at 0 h (^#^*P* < 0.0001). These findings illustrated that ADSC-Exos promoted DPC proliferation in a dose-dependent and time-dependent manner (Figure 4(b)). The scratch wound-healing assay results showed that ADSC-Exos significantly increased the wound closure area in a dose-dependent manner, and that the wound healed completely with 400 *μ*g/mL ADSC-Exos treatment at 72 h (Figure 4(c)). The results demonstrated that ADSC-Exos led to increased cell migration. As shown in Figure 4(d), the apoptosis model for DPCs was successfully established. The apoptosis rate of DPCs was elevated to 30.6% compared with that of the control group (3.66%). After treatment with 100 *μ*g/mL and 200 *μ*g/mL ADSC-Exos, we found that ADSC-Exos rescued H_2_O_2_-induced apoptosis with decreased apoptosis rates of 23.45% and 13.77%, respectively. The results depicted that ADSC-Exos mediated a protective effect against H_2_O_2_-induced apoptosis in DPCs. Taken together, these results indicated that ADSC-Exos were confirmed to significantly promote the proliferation and migration of DPCs and attenuate DPC apoptosis.

### 3.5. ADSC-Exos Alone or Combined with 5% Minoxidil Accelerated Hair Regrowth In Vivo

**Figure 5:**
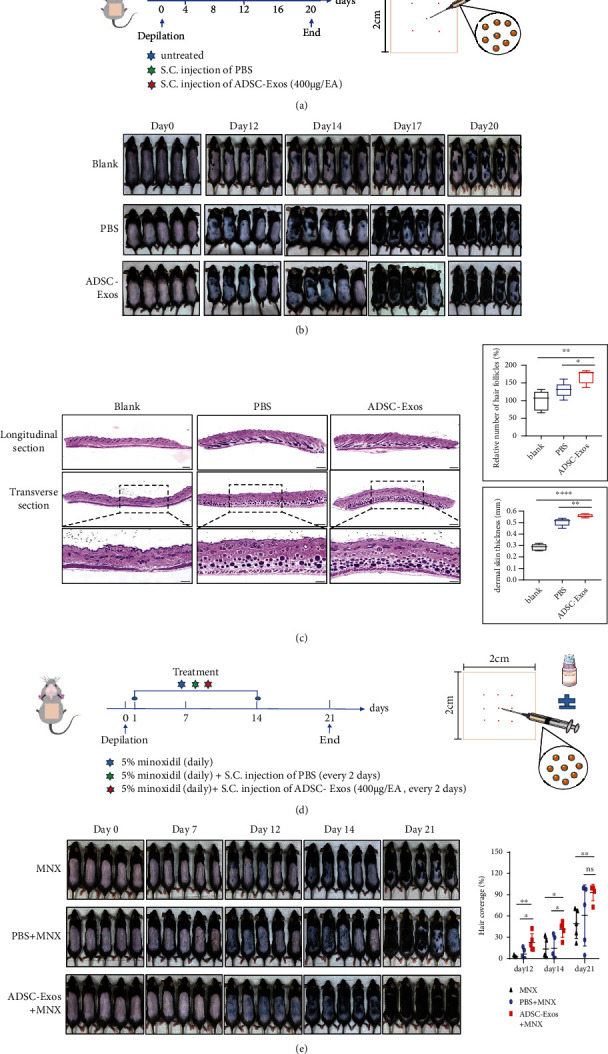
ADSC-Exos alone or combined with 5% minoxidil (MNX) accelerated hair regrowth in C57BL/6 mice. (a) Schematic representation of ADSC-Exos used alone is shown. (b) Hair growth in different groups of mice. On day 20, the mice in the ADSC-Exos group showed the best visible hair growth. (c) Histological observations of representative skin sections from dorsal skin. HF numbers and dermis thickness were calculated. Scale bar = 200 *μ*m. Cropped sections; scale bar = 100 *μ*m. (d) Schematic representation of the combination therapy with ADSC-Exos and MNX. (e) Hair growth in the MNX, PBS+MNX, and ADSC-Exos+MNX groups.

Furthermore, to investigate the effect of ADSC-Exos when ADSC-Exos were applied alone or combined with 5% minoxidil, we established a C57BL/6 mouse model. The effect of ADSC-Exos alone on hair growth was observed for 20 days and photographed on days 0, 12, 14, 17, and 20, as shown in the schematic (Figure 5(a)). On day 12, skin melanin pigmentation was observed in 3 groups, but mild hair growth was observed in the PBS group and ADSC-Exos group, and the latter grew slightly more. On days 14 and 17, there was sporadic and scattered hair growth in the blank group, but the hair growth in the PBS group and the ADSC-Exos group started to become significant, and the hair coverage area of the ADSC-Exos group was larger, especially in the first three mice. On day 20, the mice in the ADSC-Exos group showed the best visible hair growth. The hair of three mice almost completely covered the depilation area, while the hair of only two mice in the PBS group almost completely covered the depilation area, but the hair of the blank group was just partially distributed (Figure 5(b)). Collectively, the results indicated that hair growth in the ADSC-Exos group was the greatest among the 3 groups. As shown in Figure 5(c), further HE staining demonstrated that ADSC-Exos treatment differently increased the HF numbers and thickness of the dermis compared with that of the PBS group and blank group (*P* < 0.05). Thus, ADSC-Exos could slightly accelerate hair growth *in vivo*. Minoxidil is a topical drug commonly used clinically for the treatment of hair loss. Therefore, we aimed to explore whether ADSC-Exos combined with minoxidil could produce better therapeutic results than those alone. The combination of ADSC-Exos and minoxidil was tested as shown in Figure 5(d). As shown in Figure 5(e), hair growth in the ADSC-Exos+MNX group was the best among the 3 groups, and the hair coverage percentage in the ADSC-Exos+MNX group was also larger than that in the other two groups (*P* < 0.05). These results provided evidence that ADSC-Exos combined with minoxidil potently synergized in promoting hair growth compared with minoxidil application alone. These findings conclusively demonstrated that ADSC-Exos promoted hair growth *in vivo*, especially when combined with minoxidil.

### 3.6. ADSC-Exos Significantly Decreased miR-22 Levels in DPCs, Activated the Wnt/*β*-Catenin Pathway, and Potentially Exerted Anti-Inflammatory Effects

**Figure 6:**
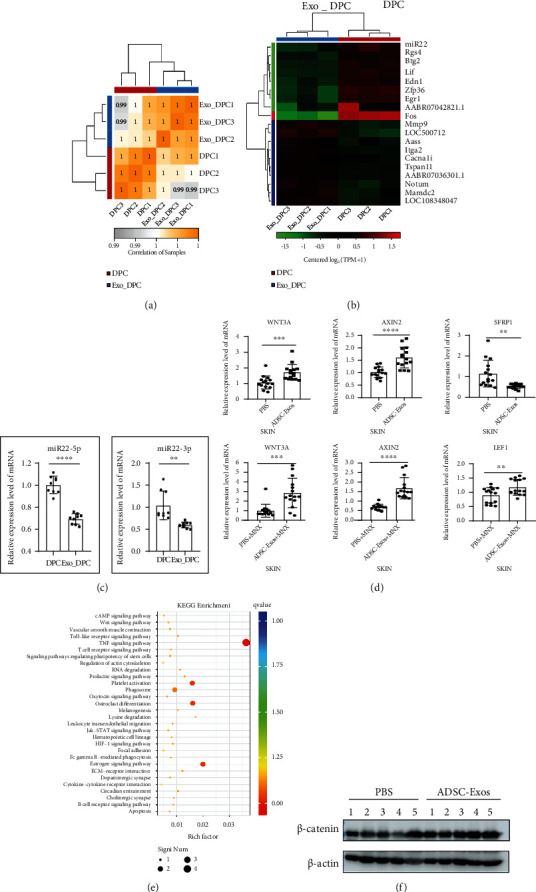
ADSC-Exos downregulated miR-22 and activated the Wnt/*β*-catenin signaling pathway, acting as potential anti-inflammatory agents. (a) Heatmap of the intersample correlation analysis showed high correlations between groups. (b) Heatmap displaying differentially expressed miR-22 in DPCs treated with ADSC-Exos (Exo_DPC) or not (DPC) by RNA-seq analysis. (c) qRT-PCR results indicated that miR-22 (miR-22-5p and miR-22-3p) levels in DPCs were downregulated after ADSC-Exos treatment. (d) Relative mRNA levels of the *β*-catenin pathway-related genes in mouse skin were detected by qRT-PCR. Compared to the control group, *WNT3A* and *AXIN2* were upregulated in the ADSC-Exos group and ADSC-Exos+MNX group. *SFRP1* was downregulated in the ADSC-Exos group, and *LEF1* was upregulated in the ADSC-Exos+MNX group. (e) Significant differential gene KEGG pathway enrichment analysis revealed that the Wnt/*β*-catenin pathway and the TNF-*α* signaling pathway were prominently regulated after ADSC-Exos treatment. (f) Western blot analysis was used to examine the protein level of *β*-catenin in mouse skin in the PBS group and ADSC-Exos group. The bands represent the results from 5 mice (from 1 to 5) in each group, respectively.

To explore the target genes of ADSC-Exos acting on DPCs, transcriptome sequencing was employed. The RNA-seq results demonstrated the groups were highly correlated (Figure 6(a)). The differential expression gene (DEG) analysis demonstrated that 10 genes were significantly upregulated, and 9 genes were markedly downregulated in the Exo_DPC group, and miR-22, a critical posttranscriptional regulator related to the hair cycle, was found to be predominantly downregulated (Figure 6(b)). Moreover, qRT-PCR results verified that miR-22 was factually expressed at lower levels (*P* < 0.01) in Exo_DPC than in DPC (Figure 6(c)).

**Figure 7:**
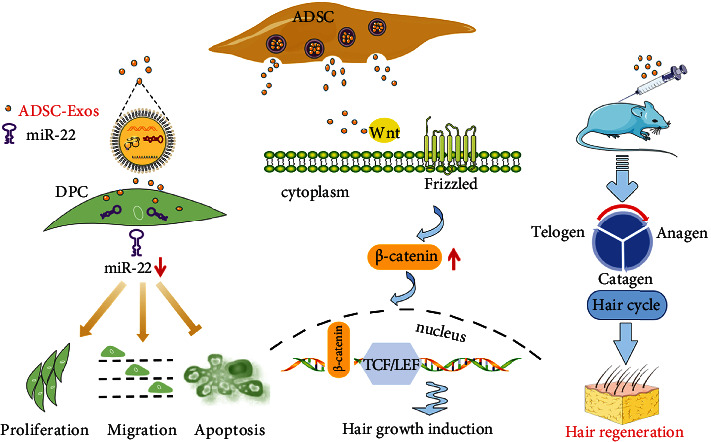
Schematic representation of the *in vitro* and *in vivo* function of ADSC-Exos. ADSC-Exos promoted DPC proliferation, migration, and inhibition of apoptosis and downregulated miR22 *in vitro*. *In vivo*, hair growth induction was initiated by accelerating hair cycle progression from telogen to anagen through the activation of the Wnt/*β*-catenin signaling pathway.

Since the Wnt/*β*-catenin signaling pathway plays an important role in hair growth *in vivo*, *β*-catenin pathway-related gene expression in mouse skin was measured by qRT-PCR. In contrast with the control group, the expression levels of *WNT3A* (*P* < 0.001) and *AXIN2* (*P* < 0.0001) in both the ADSC-Exos and ADSC-Exos+MNX groups were significantly upregulated. *LEF1* (*P* < 0.01) was upregulated in the ADSC-Exos+MNX group, and *SFRP1* (*P* < 0.01) was downregulated in the ADSC-Exos group (Figure 6(d)). The top 30 KEGG enriched pathways of DEGs (*P* < 0.05) were screened. As shown in Figure 6(e), the Wnt/*β*-catenin signaling pathway, a positive regulatory pathway of hair growth, was upregulated (*P* < 0.05) after ADSC-Exos treatment. Figure S1 in the supplementary materials showed the upregulated signaling pathways in detail. Moreover, the key proinflammatory TNF-*α* signaling pathway was prominently downregulated in DPCs after ADSC-Exos treatment (Figure 6(e)), suggesting that ADSC-Exos might play an anti-inflammatory role in hair disorders. Figure S2 in the supplementary material showed the downregulated signaling pathways in detail. Furthermore, the western blot results revealed that the protein levels of *β*-catenin were also elevated in the ADSC-Exos group skin compared to the PBS group skin (Figure 6(f)). Taken together, these results suggested that ADSC-Exos downregulated miR-22, activated the *β*-catenin pathway, and potentially acted as an anti-inflammatory agent, resulting in the promotion of hair growth (Figure 7).

## 4. Discussion

Hair loss diseases have received increasing attention in recent years. Infection, inflammation, and immune dysregulation are the main sources of immune-mediated hair loss. Depending on microbiota composition and penetration depth, scalp microbial dysbiosis may cause typical infections but may also contribute to the proinflammatory environment in chronic inflammatory scalp diseases, which can lead to hair loss. One of the most common immune-mediated alopecia is alopecia areata. There has been a lack of ideal approaches for treating alopecia due to the various limitations mentioned above. Hence, developing novel and efficient agents to treat hair loss disorders is very important. This study is aimed at exploring the function of ADSC-Exos on hair growth *in vitro* and *in vivo* for potentially immune-mediated alopecia treatment. The data demonstrated that ADSC-Exos promoted DPC proliferation, migration, inhibition of apoptosis, and hair regrowth in C57BL/6 hair-depilated mice. The effects of ADSC-Exos on DPCs and C57BL/6 hair telogen mice were first investigated in this study. The underlying mechanism may lie in the posttranscriptional regulation of miR-22, the activation of the Wnt/*β*-catenin pathway, and the potentially anti-inflammatory effect of ADSC-Exos (Figure 7).

Because of the low immunogenicity, which decreases the potential occurrence for cell rejection and graft-versus-host disease, an increasing number of studies have begun to focus on the paracrine molecules in MSCs, such as small extracellular vesicles (sEVs) [21] or exosomes [22]. Exosomes, serving as carriers of signaling molecules, can transport miRNA, mRNA, DNA, and functional proteins to recipient cells. Multiple studies have shown that MSCs, DP spheroid exosomes (DP-EVs) [15], dermal fibroblast exosomes (DF-EVs) [23], and macrophage extracellular vesicles (MAC-EVs) [24] have promising efficacy in promoting hair growth. In recent years, ADSCs have been considered one of the most promising adult MSCs in regenerative medicine because of their abundant source and yield [25]. As a cell-free derivative of ADSCs, ADSC-Exos exhibit a large number of advantages and not only bypass the limitations of clinically applying ADSCs but also possess the following merits: facile storage, transport, and commercial production.

As an important mesenchymal component of HFs, DPCs play a central role in hair regeneration by participating in epidermal-mesenchymal interactions [9]. Hair growth is accompanied by the proliferation and migration of DPCs. Using fluorescently Dil-labeled ADSC-Exos, we confirmed they were taken up by DPCs. Then, we found that ADSC-Exos also promoted the proliferation and migration of DPCs. Catagen in the hair cycle is considered apoptosis-driven regression of HFs [26]. To examine whether ADSC-Exos possess an antiapoptotic effect, flow cytometric experiments were employed. The results suggested that ADSC-Exos could rescue H_2_O_2_-induced apoptosis in DPCs. These findings were in accordance with a previous study on cutaneous wound healing [27]. To further study the effect of ADSC-Exos on hair regrowth *in vivo*, we selected C57BL/6 mice as an ideal animal model. The dorsal hair of C57BL/6 mice has a time-synchronized growth cycle, in which the color of depilated dorsal skin transforms from pink in telogen to black in anagen [28, 29]. In our study, ADSC-Exos alone or combined with minoxidil were injected subcutaneously into dorsal skin in the telogen phase. As expected, the hair growth in the ADSC-Exos-treated group with or without the combination of minoxidil was visibly the best compared to that of the control groups. During the anagen period, hair follicles are typically characterized by an increased number of HFs and dermis thickness [30, 31]. In our study, HE staining revealed more HFs, and a greater dermis thickness was detected in the ADSC-Exos treatment group than in the other groups.

miR-22 is a highly conserved microRNA that is highly expressed in the heart [32]. Recently, it has been shown that miR-22 is a critical posttranscriptional regulator of the hair cycle that promotes anagen-to-catagen transition, leading to HF regression [33]. In our study, RNA-seq revealed that miR-22 was expressed at low levels in DPCs after ADSC-Exos incubation. Then, we further verified this result by qRT-PCR. Wnt/*β*-catenin signaling is an important pathway involving hair growth [34]. Whether ADSC-Exos promote hair growth through the activation of the Wnt/*β*-catenin pathway is unclear. In our study, RNA-seq demonstrated that the Wnt/*β*-catenin pathway was upregulated after ADSC-Exos treatment in DPCs. qRT-PCR and western blot results also demonstrated that the Wnt/*β*-catenin pathway in the skin of the ADSC-Exos treatment group was activated. These results suggested that ADSC-Exos initiated the activation of the Wnt/*β*-catenin pathway. Moreover, RNA-seq results indicated that ADSC-Exos have potential anti-inflammatory effects with the downregulation of the TNF-*α* signaling pathway. Collectively, we illustrated that ADSC-Exos downregulated miR-22, activated the Wnt/*β*-catenin signaling pathway, and potentially acted as an anti-inflammatory agent, ultimately resulting in hair regrowth. An in-depth study of crucial and functional molecules in ADSC-Exos needs to be performed and verified in the future. Overall, we shed light on the notion that ADSC-Exos promote hair regrowth by downregulating miR-22 expression and the TNF-*α* signaling pathway, along with Wnt/*β*-catenin signaling activation.

## 5. Conclusion

In the present study, we demonstrated that ADSC-Exos promoted proliferation, migration, and apoptosis remission in DPCs. The *in vivo* mouse experiments also supported the observation that ADSC-Exos played a critical role in hair regrowth, which was consistent with the *in vitro* results. The effects of ADSC-Exos on DPC biological function and hair growth in C57BL/6 hair telogen mice were investigated for the first time in this study. The underlying mechanism may involve miR-22 downregulation in posttranscriptional regulation, the activation of the Wnt/*β*-catenin signaling pathway, and the potentially anti-inflammatory effect. In conclusion, this study provides the potential for developing ADSC-Exos as promising therapeutic EVs for a cell-free treatment strategy of immune-mediated hair loss in the future.

## Data Availability

The datasets generated or analyzed during the current study are available from the corresponding authors on reasonable request.

## Supplementary Materials

Supplementary Materials Table S1: primer sequences for qPCR. Figure S1: the upregulated signaling pathways in DPCs after ADSC-Exos treatment by KEGG pathway enrichment analysis. Figure S2: the downregulated signaling pathways in DPCs after ADSC-Exos treatment by KEGG pathway enrichment analysis.
