# Supplementary material for: Exosomes Secreted from Adipose-Derived Stem Cells Are a Potential Treatment Agent for Immune-Mediated Alopecia
Source: J Immunol Res. 2022 Feb 3;2022:7471246. doi: 10.1155/2022/7471246 (PMC8831060; doi:10.1155/2022/7471246)
Supplement: Supplementary Materials — Table S1: primer sequences for qPCR. Figure S1: the upregulated signaling pathways in DPCs after ADSC-Exos treatment by KEGG pathway enrichment analysis. Figure S2: the downregulated signaling pathways in DPCs after ADSC-Exos treatment by KEGG pathway enrichment analysis. [file 7471246.f1.zip › Table S1.pdf]

Table S1. Primer sequences for qPCR

Species	Primer Name	Forward	Reverse
Mouse	WNT3A	AACTGCACCACCGTCAGCAACA	AGCGTGTCACTGCGAAAGCTA
	AXIN2	ATGGAGTCCCTCCTTACCGCAT	GTTCCACAGGCGTCATCTCCTT
	LEF1	ACTGTCAGGCGACACTTCCATG	GTGCTCCTGTTTGACCTGAGGT
	SFRP1	CAATACCACGGAAGCCTCTAAGC	GCAAACTCGCTTGCACAGAGATG
	β-actin	GGCTGTATTCCCCTCCATCG	CCAGTTGGTAACAATGCCATGT
Rat	SOX2	GCACATGAACGGCTGGAGCAACG	TGCTGCGAGTAGGACATGCTGTAGG
	β-actin	TTCTTGCAGCTCCTCCGTCG	AGTCCTTCTGACCCATACCCA
	Mir22-5p	ACACTCCAGCTGGGag ttcttcagtg gc	TGGTGTCGTGGAGTCG
	Mir22-3p	ACACTCCAGCTGGGaagc tgccagttga a	TGGTGTCGTGGAGTCG
	U6	CTCGCTTCGGCAGCACA	AACGCTTCACGAATTTGCGT
	Mir22-5pRT	CTCAACTGGTGTCGTGGAGTCGGCAATTCAGTTGAGTAAAGCTT	
	Mir22-3pRT	CTCAACTGGTGTCGTGGAGTCGGCAATTCAGTTGAGACAGTTC	
