# Supplementary figures and images for: Exosomes Secreted from Adipose-Derived Stem Cells Are a Potential Treatment Agent for Immune-Mediated Alopecia
Source: J Immunol Res. 2022 Feb 3;2022:7471246. doi: 10.1155/2022/7471246 (PMC8831060; doi:10.1155/2022/7471246)

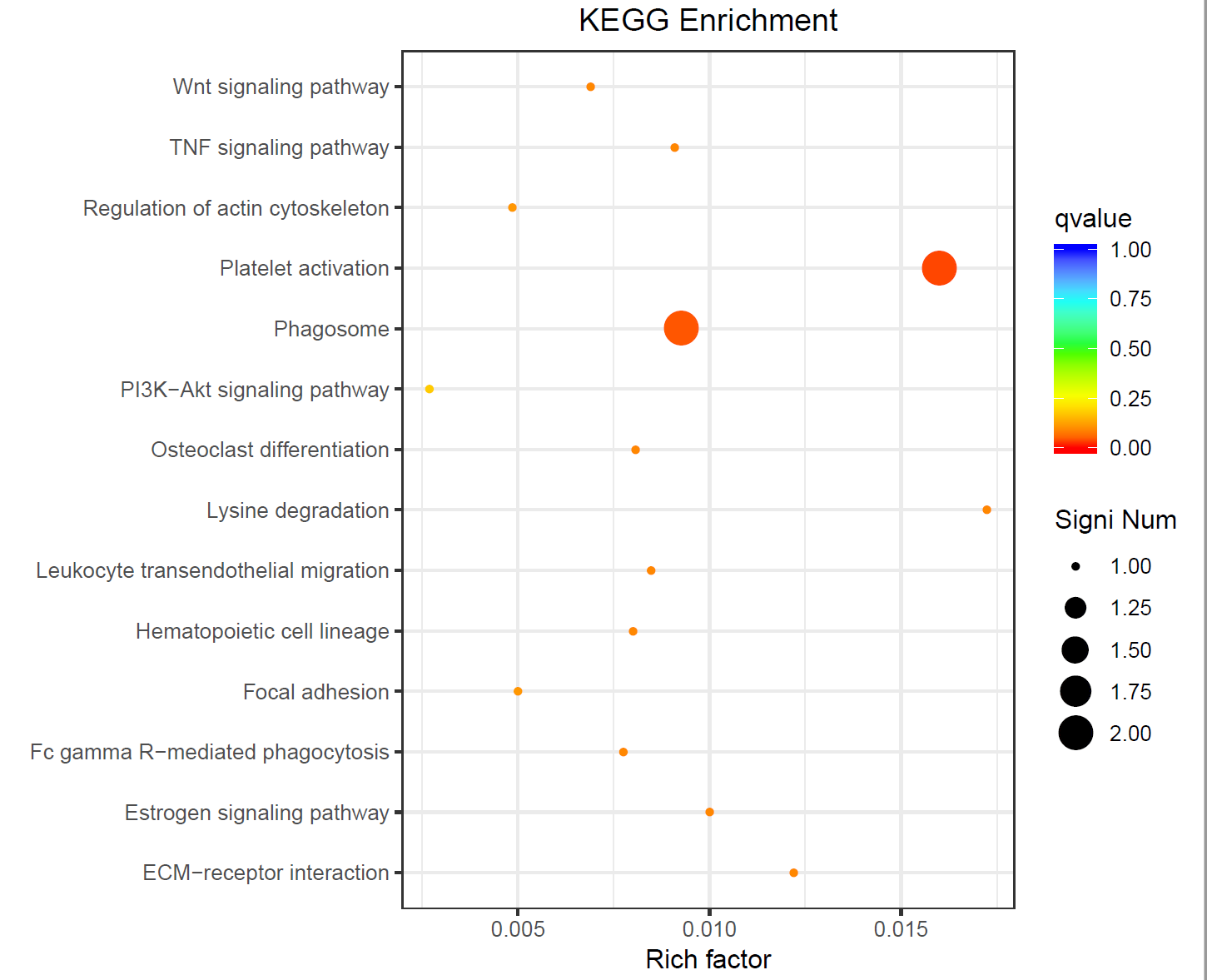

Supplement: Supplementary Materials — Table S1: primer sequences for qPCR. Figure S1: the upregulated signaling pathways in DPCs after ADSC-Exos treatment by KEGG pathway enrichment analysis. Figure S2: the downregulated signaling pathways in DPCs after ADSC-Exos treatment by KEGG pathway enrichment analysis. [file 7471246.f1.zip › Figure S1 (Exo_DPC_vs_DPC.up_KEGG_enrichment_scatter).png]

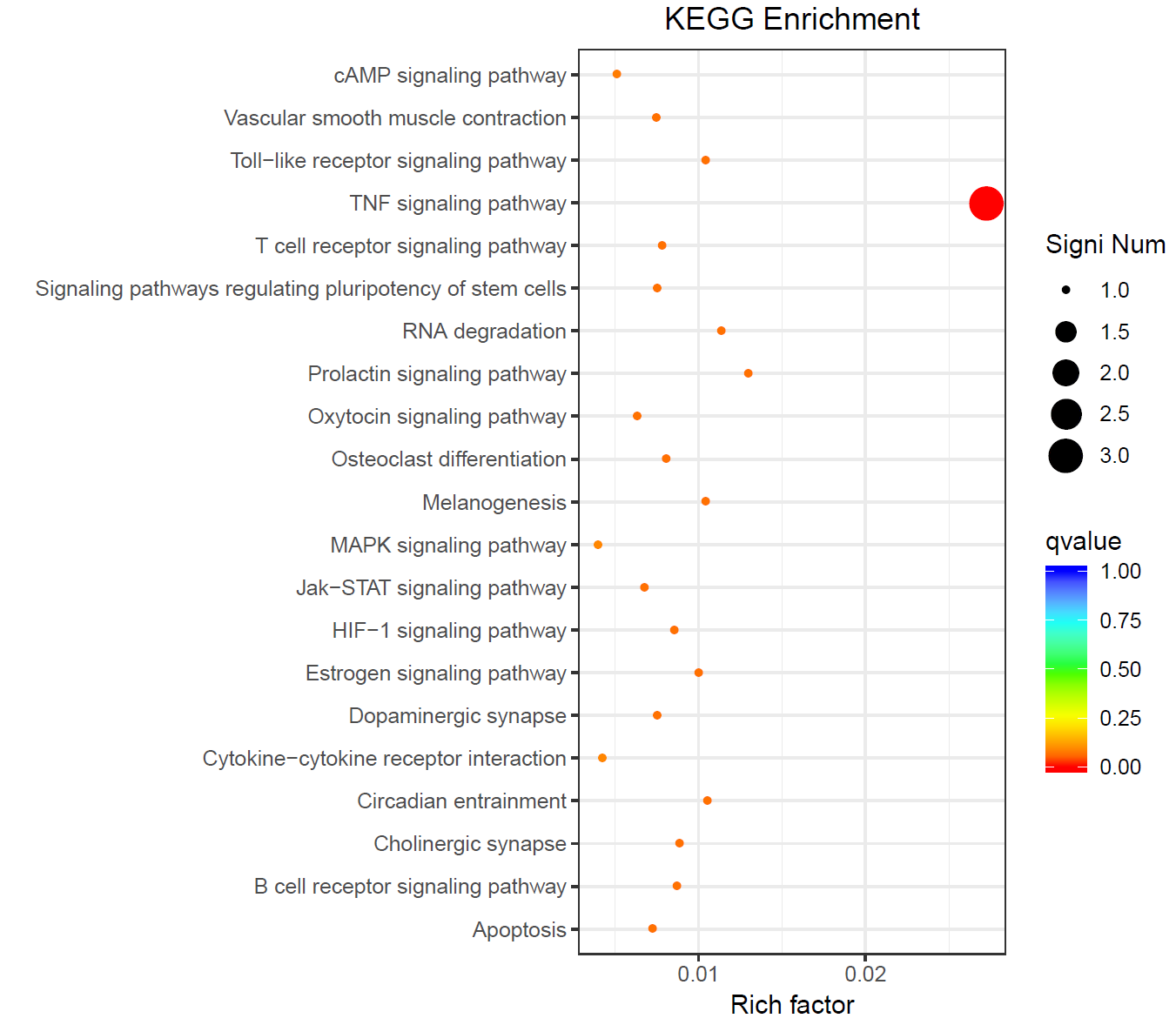

Supplement: Supplementary Materials — Table S1: primer sequences for qPCR. Figure S1: the upregulated signaling pathways in DPCs after ADSC-Exos treatment by KEGG pathway enrichment analysis. Figure S2: the downregulated signaling pathways in DPCs after ADSC-Exos treatment by KEGG pathway enrichment analysis. [file 7471246.f1.zip › Figure S2 (Exo_DPC_vs_DPC.down_KEGG_enrichment_scatter).png]
